# Spooky Interaction at a Distance in Cave and Surface Dwelling Electric Fishes

**DOI:** 10.3389/fnint.2020.561524

**Published:** 2020-10-22

**Authors:** Eric S. Fortune, Nicole Andanar, Manu Madhav, Ravikrishnan P. Jayakumar, Noah J. Cowan, Maria Elina Bichuette, Daphne Soares

**Affiliations:** ^1^Biological Sciences, New Jersey Institute of Technology, Newark, NJ, United States; ^2^Department of Mechanical Engineering, Johns Hopkins University, Baltimore, MD, United States; ^3^Departamento de Ecologia e Biologia Evolutiva, Universidade Federal de São Carlos, São Carlos, Brazil

**Keywords:** gymnotiformes, weakly electric fish, troglobitic, epigean, envelope, cavefish, jamming avoidance response, diceCT

## Abstract

Glass knifefish (*Eigenmannia*) are a group of weakly electric fishes found throughout the Amazon basin. Their electric organ discharges (EODs) are energetically costly adaptations used in social communication and for localizing conspecifics and other objects including prey at night and in turbid water. Interestingly, a troglobitic population of blind cavefish *Eigenmannia vicentespelea* survives in complete darkness in a cave system in central Brazil. We examined the effects of troglobitic conditions, which includes a complete loss of visual cues and potentially reduced food sources, by comparing the behavior and movement of freely behaving cavefish to a nearby epigean (surface) population (*Eigenmannia trilineata*). We found that the strengths of electric discharges in cavefish were greater than in surface fish, which may result from increased reliance on electrosensory perception, larger size, and sufficient food resources. Surface fish were recorded while feeding at night and did not show evidence of territoriality, whereas cavefish appeared to maintain territories. Surprisingly, we routinely found both surface and cavefish with sustained differences in EOD frequencies that were below 10 Hz despite being within close proximity of about 50 cm. A half century of analysis of electrosocial interactions in laboratory tanks suggest that these small differences in EOD frequencies should have triggered the “jamming avoidance response,” a behavior in which fish change their EOD frequencies to increase the difference between individuals. Pairs of fish also showed significant interactions between EOD frequencies and relative movements at large distances, over 1.5 m, and at high differences in frequencies, often >50 Hz. These interactions are likely “envelope” responses in which fish alter their EOD frequency in relation to higher order features, specifically changes in the depth of modulation, of electrosocial signals.

## 1. Introduction

Gymnotiformes are a group of nocturnal fishes characterized by a suite of adaptations that allow them to localize conspecifics (Davis and Hopkins, 1988; Crampton, 2019) and capture prey (Nelson and MacIver, 1999) in complete darkness. These fishes produce weak electric fields, typically <100 mV/cm (Assad et al., 1998), using an electric organ located along the sides of the animal and in the tail (Heiligenberg, 1991; Markham, 2013). This electric field, known as the electric organ discharge or EOD, is detected using specialized electroreceptors embedded in the skin (Metzen et al., 2017). These receptors encode modulations generated through interactions with the electric fields of conspecifics and by nearby objects. This system provides a mechanism for communication among conspecifics and for the detection and characterization of prey and other salient environmental features (Nelson and MacIver, 1999; Pedraja et al., 2018; Crampton, 2019; Yu et al., 2019) at night and in turbid water that reduce visual cues.

On one hand, the nocturnal life histories of Gymnotiform species, facilitated by their electrosensory systems, make them well-suited for life in caves. On the other hand, caves are often poor in nutrients: the generation of EODs is energetically costly, consuming up to one quarter of an individual's energy budget (Salazar et al., 2013; Markham et al., 2016). Interestingly, a single species of Gymnotiform fish, *Eigenmannia vicentespelaea*, has been discovered in a cave system in central Brazil (Triques, 1996; Bichuette and Trajano, 2006, 2017). These fish exhibit features that are commonly found in species adapted to life in caves, including reduced pigmentation and reduction and/or elimination of the eyes (Culver and Pipan, 2019). The population is estimated to be around only 300 individuals (Bichuette and Trajano, 2015).

**Figure 1:**
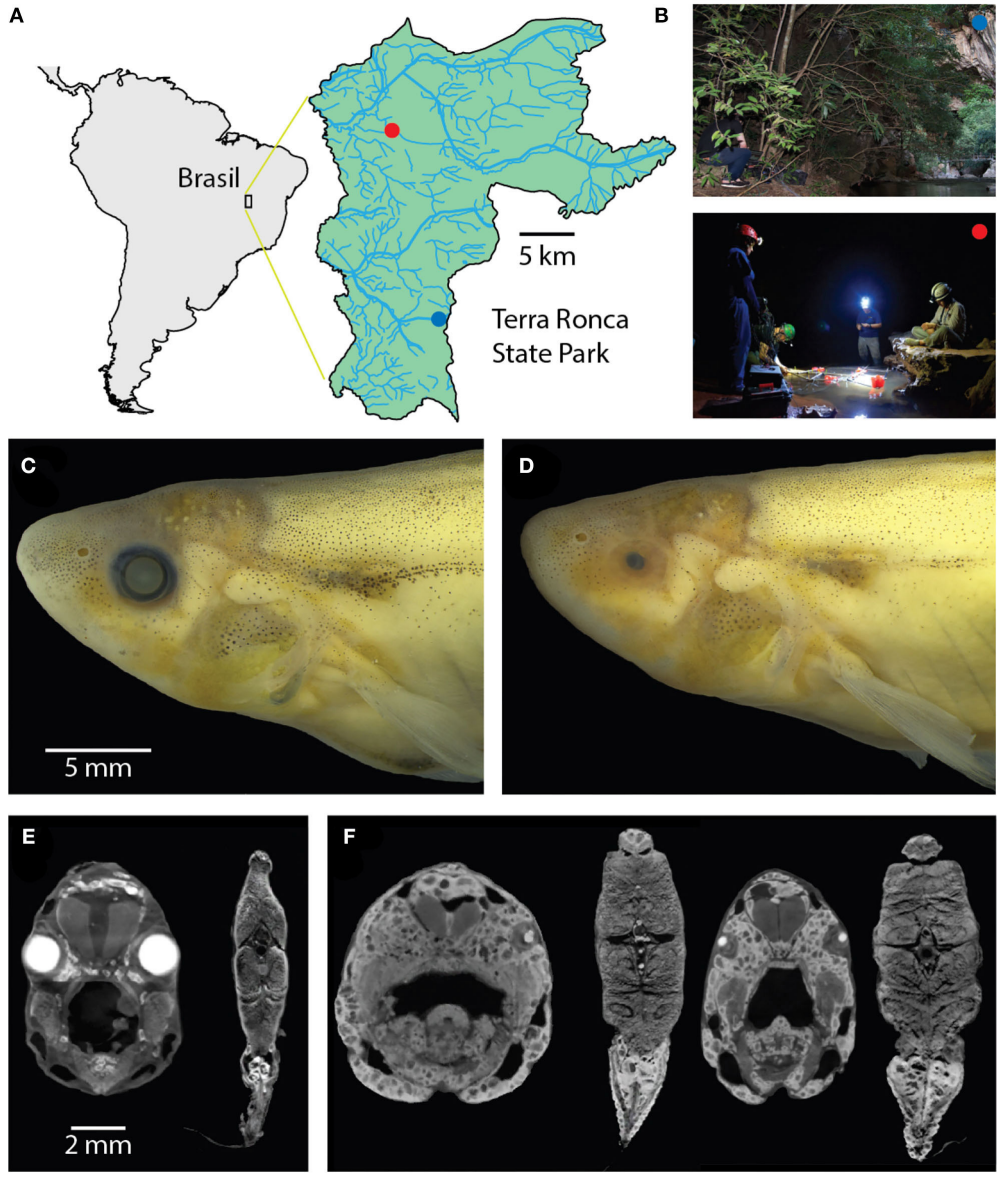
Surface and cave *Eigenmannia*. **(A)** Study sites are in a clear water system in the Rio da Lapa karst region in Goiás, Brazil. **(B)** Top, the surface *Eigenmannia* site is in the entrance of the Rio da Lapa cave. Bottom, the cave *Eigenmannia* are found in the São Vicente II cave. **(C)** Surface *Eigenmannia* have well-developed eyes and distinctive markings. **(D)** Cave *Eigenmannia* have poorly developed or missing eyes and reduced pigmented features. diceCT imaging reveals the differences in eye sizes and a potential difference in the relative size of electric organs. **(E)** Coronal sections through the head and mid-body of a surface fish and **(F)** two cavefish (dorsal is up). Large, bright cells in the caudoventral coronal sections appear relatively larger in the cave vs. surface species.

**Figure 2:**
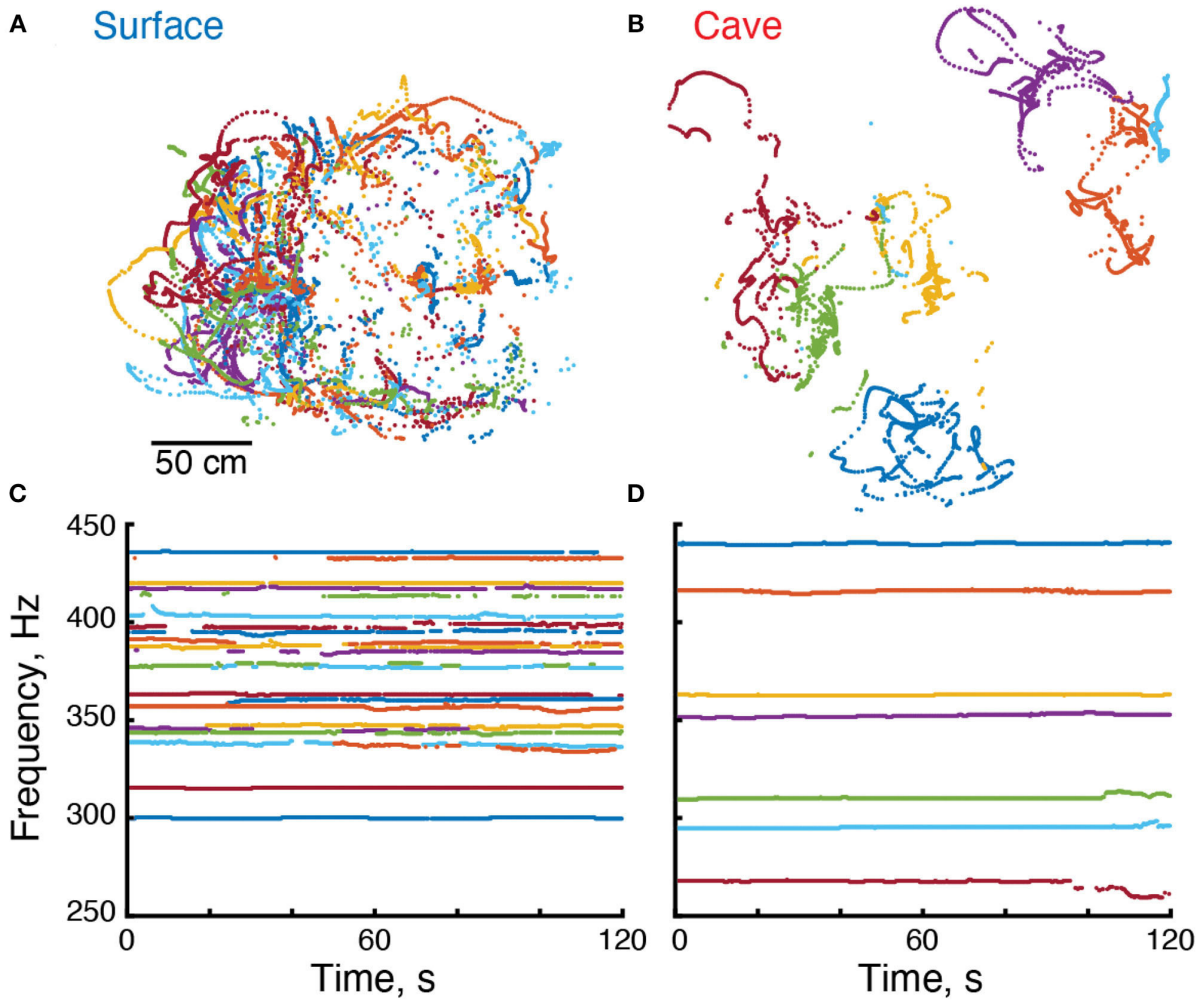
Electrical and locomotor behavior of *Eigenmannia*. **(A,B)** Positions of surface and cave *Eigenmannia* over a period of 120 s. **(C,D)** EOD frequencies of these fish. Each color represents a unique fish in each recording.

To discover the potential consequences of adaptation for troglobitic life on electrosensory behavior, we compared the electric behavior and movement of the cavefish *Eigenmannia vicentespelea* to nearby epigean (surface) relatives, *Eigenmannia trilineata*, that live in the same river system (Figure 1). We used a recently developed approach for characterizing electric behaviors and locomotor movements of weakly electric fishes in their natural habitats (Madhav et al., 2018; Henninger et al., 2020). This approach, which uses a grid of electrodes placed in the water, permits an estimation of features of the electric field of each fish and an analysis of their concurrent movement (Figure 2).

We looked for differences between surface and cavefish populations in electrogenic behaviors and movement, and in previously-described electrosocial behaviors. Fish in the genus *Eigenmannia* produce quasi-sinusoidal electric signals that are maintained at frequencies between about 200 and 700 Hz (Heiligenberg, 1991). Individual *Eigenmannia* change their electric field frequencies in response to electrosocial signals produced by nearby conspecifics. The best described of these behaviors is the “Jamming Avoidance Response” (JAR) in which individuals raise or lower their electric field frequency to avoid differences of less than about 10 Hz (Watanabe and Takeda, 1963; Heiligenberg, 1991; Madhav et al., 2013). *Eigenmannia* also exhibit “envelope responses” in which individuals change their electric field frequencies in relation to relative movement between individuals, which is encoded in the amplitude envelope of their summed electric fields (Metzen and Chacron, 2013; Stamper et al., 2013; Huang and Chacron, 2016; Thomas et al., 2018). Envelope responses can also occur in groups of three or more fish when the pairwise differences of their electric field frequencies are close, within 1–8 Hz, of one another (Stamper et al., 2012). Finally, weakly electric fishes produce a variety of transient frequency and amplitude modulations, including “chirps,” with durations on the order of 10s of milliseconds to 10s of seconds. These signals have roles in aggression, dominance hierarchies, and in other social interactions (Hupe and Lewis, 2008; Walz et al., 2013; Allen and Marsat, 2019; Metzen, 2019).

## 2. Methods

These observational studies were reviewed and approved by the animal care and use committee of Rutgers University/New Jersey Institute of Technology, and follow guidelines for the use of animals in field research established by the National Research Council. Field research permits in Brazil were granted by the ICMBio and SEMARH/SECIMA.

### 2.1. Study Sites

The study sites were located in Terra Ronca State Park (46° 10′–46° 30′ S, 13° 30′–13° 50′ W), in the Upper Tocantins river basin, state of Goiás, central Brazil (Figure 1A). We measured the electric behavior of the cavefish *Eigenmannia vicentespelaea* in the São Vicente II cave (13° 58′37″ S, 46° 40′04″ W) in October of 2016 (Figure 1B). The electric behavior of the epigean species, *Eigenmannia trilineata*, was measured in the Rio da Lapa at the mouth of the Terra Ronca cave (13° 38′44″ S; 46° 38′ 08″ W) in April of 2016 (Figure 1B). These streams have moderate water currents, clear water with conductivity below 20 μS, and the substrate is composed of sand, rocks, and boulders.

### 2.2. Anatomy

Four alcohol fixed specimens from the collection at the Universidade Federal de São Carlos (Dr. Bichuette) were submerged in 11.25 Lugol's iodine (I2KI) solution for up to 36 h prior to diffusable iodine based contrast enhanced computer tomography (DiceCT). Stained specimens were removed from Lugol's solution, rinsed in water to remove excess stain and sealed in rubber sleeves to prevent dehydration. Samples were then loaded into 50 mL polypropylene centrifuge tubes for scanning.

Stained and unstained specimens were scanned at the Core Imaging Facility of the American Museum of Natural History (New York, NY), using a 2010 GE Phoenix v∣tome∣x s240CT high resolution microfocus computed tomography system (General Electric, Fairfield, CT, USA). DiceCT scanning permits visualization of soft tissue details of the head and the body. Scans were made at 125 kV, with an exposure time of 60 s. Voxel sizes was 20.0–25.9 μm. Volume reconstruction of raw X-ray images were achieved using a GE Phoenix datos∣x.

### 2.3. Recordings of Electric Behavior at Field Sites

*Eigenmannia* were initially identified and located using hand-held single-electrode probes with a custom amplifier/speaker system. This river permits direct visualization of the animals—the water is sufficiently clear and free of debris to see fish by eye from above the surface of the water, and for underwater photography when the fish are swimming in open water (Hero Cam, GoPro 3, USA, see Supplementary Videos 1, 2).

Electric recordings were made using a grid of active electrodes (50 cm spacing) (Madhav et al., 2018). For measurements of the epigean fish, an array of eight electrodes was placed along the edges of the Rio da Lapa stream after sundown when the fish were active. In the São Vicente II cave, an array of 16 electrodes was placed in eddies and side pools along the primary stream. The flow at the center of the stream was too strong for the grid array. Unfortunately, we were unable to use the larger grid on our second visit to the surface site due to a concurrent religious festival. As a result, the maximum XY range of the surface grid is about 100 cm diameter smaller than the cave grid. In all other respects, the measurements from both grids are identical.

We used an algorithm (Madhav et al., 2018) (code available at doi: 10.7281/T1/XTSKOW) to identify each *Eigenmannia* using its time-varying fundamental frequency (Figure 2). Position and pose were calculated in relation to the distribution of power at each EOD frequency across the grid of electrodes. In these recordings, which were made in shallow water of no more than 40 cm depth using a planar array of electrodes, the position estimates were restricted to the XY-plane (Figures 2A,B). We calculated an estimate of the strength of the electric field for each fish. Fish were considered to be ideal current dipoles—a source-sink pair of equal but time varying strength *I*(*t*), separated by a small distance *d*. The electric current dipole moment for the fish is defined as *p* = *Id* which has the units of Ampere-meter or “Am”.

Continuous recording sessions using the grid were made both at the cave site (*N* = 14) and surface site (*N* = 5). Each recording had a duration of at least 600 s, while others were over 1,200 s. Intervals between recording sessions ranged from 5 min to several hours. Because fish could not be tracked between recording sessions, it is likely that some individual fish were measured across sessions. The position and EOD frequency data were analyzed, unless otherwise described, in 300 s duration non-overlapping epochs.

### 2.4. Analysis of Position and EOD Frequency Data

All analyses were conducted using custom scripts in Matlab (Mathworks, Natick MA). These data and scripts are publically available (https://web.njit.edu/~efortune/Brasil). We estimated the XY region of movement for each fish for each epoch using a minimum convex polygon fitted to its positions. We then calculated the pairwise overlap between each convex polygon.

To assess the relations between pairs of EOD frequencies and their relative movement, we calculated Pearson correlations between (1) instantaneous distance between pairs of fish (distance) and (2) instantaneous difference in EOD frequency (dF). Euclidean distances between pairs of fish were computed as a function of time (4.9 measurements per second) during each 300 s epoch. dF was calculated as the absolute value of the difference in EOD frequencies of the pair of fish. The Pearson correlation was calculated between distance and dF for each 300 s epoch. These “dF/distance correlations” ranged from −0.93 to 0.90. Negative Pearson correlations represent an inverse relation between dF and distance, where positive Pearson correlations represent direct correlations between them. To estimate the rate of spontaneous correlations between dF and distance, we shuffled (Matlab randperm) the epochs of dF and distance measurements and again calculated Pearson correlations. We then compared the distribution of correlations in the shuffled data to the correlations in the original data.

## 3. Results

### 3.1. General Observations

*Eigenmannia* at both the surface and cave sites were found in clear water streams with rock and sand substrates (Bichuette and Trajano, 2003, 2006, 2015, 2017). At the surface site, we observed a marked diurnal modulation of behavior. During the day, surface fish were found alone or in groups along the banks of the Rio da Lapa, typically below or around boulders and rocks. The grid system was not used to make recordings of surface fish during the day due to a local festival. Nevertheless, we used hand held probes to examine the distribution of surface *Eigenmannia* during the day, and the distribution appeared, by ear, to be similar to that described previously at sites in Ecuador (Tan et al., 2005). Unlike in previous measurements at other study sites (Stamper et al., 2010), no other Gymnotiform species were detected in our short survey. At night, we observed surface *Eigenmannia* swimming in open water in the center of the stream, typically near the bottom.

Using flashlights, we observed surface fish foraging in sandy substrates at night. Foraging behavior included hovering with slow forward or backward swimming punctuated by strikes into the substrate (see Supplementary Video 1). These strikes involved tilting of the head and body downwards with a rapid forward lunge to drive the mouth into the sand a few millimeters. These strikes differ from feeding behavior described in *Apteronotus albifrons* in which fish captured freely swimming *Daphnia*, typically from below the prey (Nelson and MacIver, 1999). We observed groups of *Eigenmannia* simultaneously foraging with inter-fish distances on the order of 10s of centimeters. These distances suggest that fish experience significant ongoing electrosensory interference from conspecifics. At other locations, we visually observed single fish foraging while swimming rather than hovering at a particular location.

Not surprisingly, we did not observe diurnal modulation of behavior in cave *Eigenmannia*. Cavefish were observed along the banks of the stream and in small eddies and pools. Fish were alone or in small groups of up to 10 individuals. Cavefish retreated to crevices and spaces within boulders and rocks when disturbed, forming temporary aggregates of individuals (see Supplementary Video 2). Videos show eyeless cavefish orienting face-to-face during social interactions. Such movements, in the absence of visual cues, are controlled, at least in part, using electrosensory signals. The distinctive substrate foraging behavior routinely seen in the surface fish was not observed in these cavefish.

### 3.2. Morphology

We observed that surface *Eigenmannia* had large eyes that were circumferential and of the same size (Figures 1C,E) whereas the cavefish had eyes in various states of degeneration, from microphthalmic to completely absent (Figures 1D,F). A preliminary review of CT scans of four fish showed potential differences in the size of the electric organ (Figures 1E,F), but additional material will be necessary for quantitative analysis of electric organ structure and physiology.

A prior study (Bichuette and Trajano, 2006) found that the surface *Eigenmannia* are smaller than the cavefish: mean length of the snout to the end of the anal fin base was reported to be 8.45 cm (sd = 2.67) in surface fish and 11.1 cm (sd = 2.47) in cavefish. Size is important as it likely impacts the strength of electric fields: larger fish typically can generate larger currents in their electric organs. Fish were not captured for similar measurements in the present study.

### 3.3. Electric Field Amplitudes

**Figure 3:**
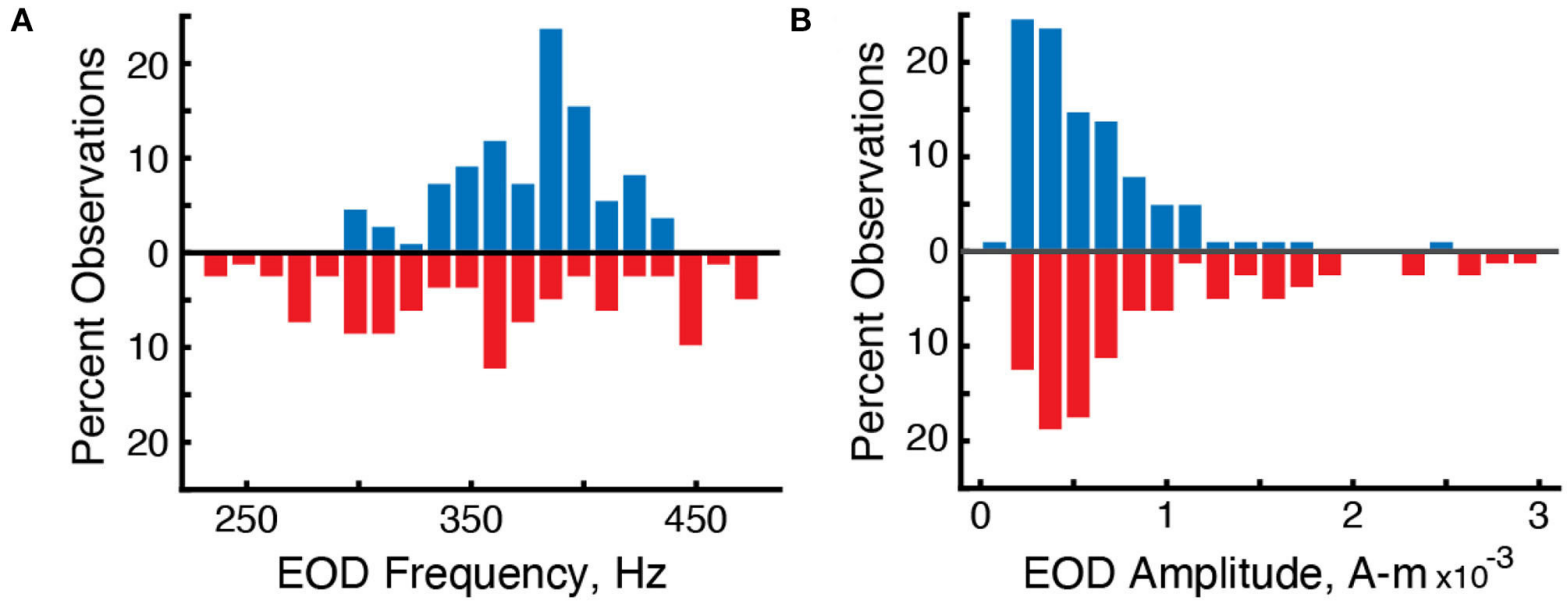
Differences in the distribution of EOD frequencies and amplitudes in surface fish (blue, up) and cavefish (red, down). **(A)** Percent observations of EOD frequencies for surface fish and cavefish. **(B)** Percent observations of EOD amplitudes.

We used the grid recording system to estimate the strength of each individual's electric field using Fourier analysis. The mean strength of the electric fields (Ampere-meter, “A-m”) of surface fish was 6.5 × 10^-4^ A-m (*n* = 110 EOD frequencies in 5 recordings) and 9.66 × 10^-4^ A-m for cavefish (*n* = 82 EODs in 14 recordings) (Figure 3B). The strengths of surface fish were significantly lower than cavefish (Wilcoxon rank sum, two-sided, *z* = 2.98, *p* = 0.0029). The increase in EOD amplitude may be an energetically costly adaptation (Markham et al., 2016) to life in this cave: larger EOD amplitudes would increase the ability to detect objects and capture prey (Nelson and MacIver, 1999) in the absence of visual cues.

### 3.4. Electric Field Frequencies

We used the grid recording system to calculate the EOD frequency of each fish within the grid (Figure 2). EOD frequencies of surface fish were between 299.9 and 435.6 Hz (*n* = 110 EODs in five recordings) whereas EOD frequencies of cavefish were between 230.0 and 478.6 Hz (*n* = 82 EODs in 14 recordings) (Figure 3A). The mean and median frequencies of surface fish were 375.8 and 382.0 Hz, and for cavefish were 356.6 and 360.8 Hz. The distribution of EOD frequencies of surface and cavefish are significantly different (Wilcoxon sign-rank, two-sided, *z* = 2.57, *p* = 0.0100).

We observed variations in EOD frequencies that likely include social signals, such as chirps (Figures 2C,D). Although a detailed description of these social signals is beyond the scope of this report, we calculated the standard deviation of each EOD frequency as a simple proxy for the rate of production of social signals. Variation in EOD frequencies were calculated over 300 s duration epochs. The EOD frequencies of surface fish had a mean standard deviation of 0.64 Hz (184 epochs from 97 fish). Variation in EOD frequencies of cavefish had a mean standard deviation of 1.11 Hz (257 epochs from 72 fish). The variation of EOD frequencies in cavefish was significantly different from surface fish (Wilcoxon rank sum, two-sided, *z* = 4.97, *p* = 0.000001). We expect the variability of EOD frequencies in surface fish will differ during daylight hours, when the fish are hiding along the shores of the rivers, in relation to night hours when the fish are active. Additional recordings made during the day are needed to test this hypothesis.

The electric fields of some species of Gymnotiform fishes have been shown to have diurnal modulations in amplitude and frequency content (Stoddard et al., 2006; Markham et al., 2009; Sinnett and Markham, 2015; Migliaro et al., 2018). We did not observe diurnal modulation of EOD frequencies in cave *Eigenmannia*. Because we did not make daytime recordings at the surface site, we do not know whether or not such modulations are present in the surface *Eigenmannia*.

We recorded preliminary data which suggest that the fish's own movement can contribute to modulation of EOD frequencies. We placed three surface *Eigenmannia* in tubes as part of a calibration of the grid system. These tubes restricted the animal's movement to a few centimeters in the grid. Those fish had significantly less variability in their EOD frequencies than the freely-moving fish swimming around them (mean = 0.38 Hz, 29 epochs from three fish; Wilcoxon rank sum, two-sided, *z* = −4.40, *p* = 0.000011).

### 3.5. Differences in EOD Frequencies

**Figure 4:**
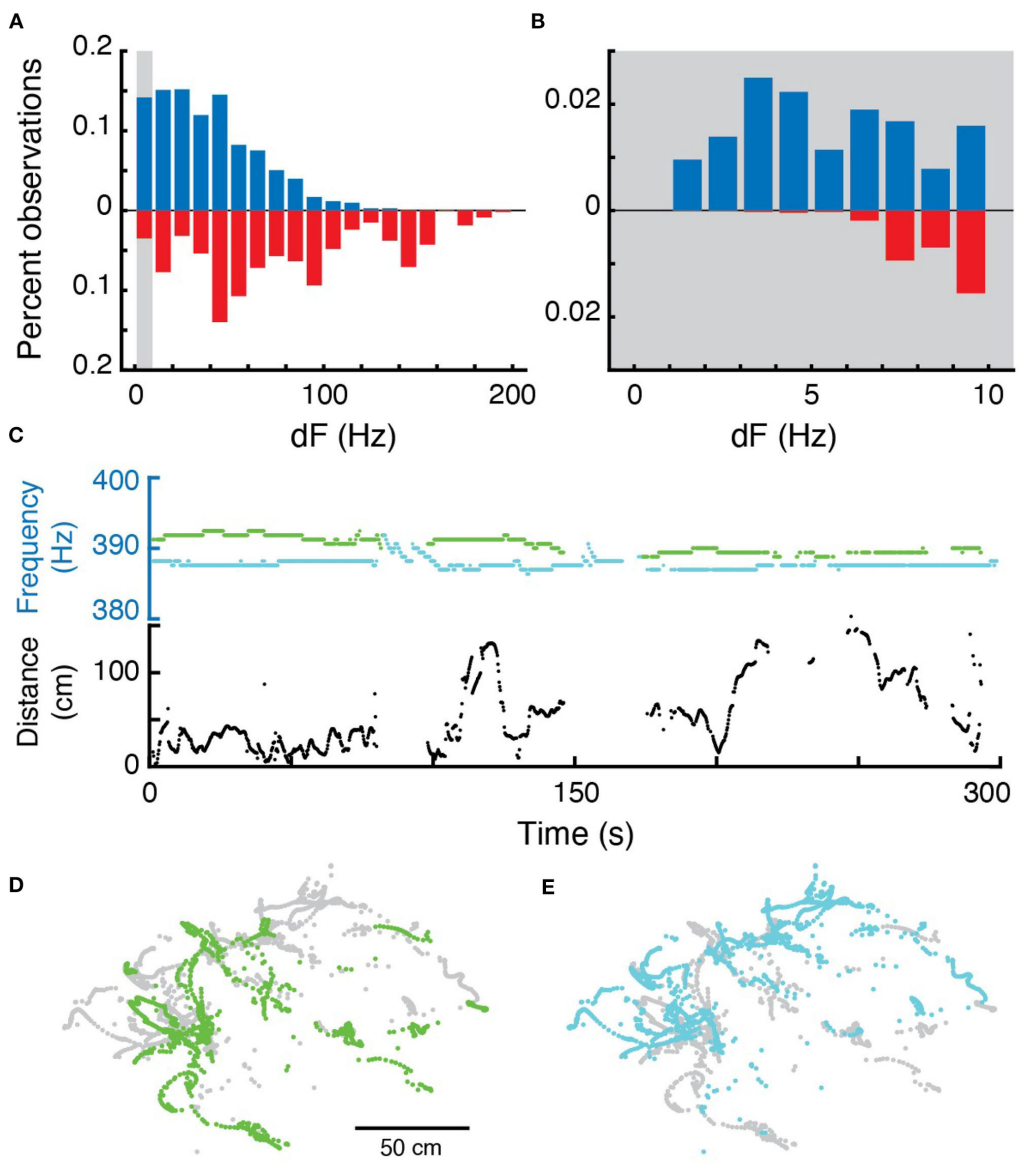
Differences in EOD frequencies. **(A)** Percent observations of dFs for surface fish (blue, up) and cavefish (red, down). Gray bar highlights the low frequency region shown in **(B)**. **(B)** Same as **(A)**, but for dF frequencies of 10 Hz and below. **(C)** Example 300 second epoch of two fish with sustained low-frequency dF. Top, EOD frequencies each fish; bottom Euclidian distance over time. **(D,E)** X-Y positions of each fish during this epoch.

The JAR is a behavior that, in laboratory settings, reduces the likelihood that pairs of fish will have a difference in EOD frequency (dF) of <10 Hz (Heiligenberg, 1991). For each pair of EOD frequencies, the mean dF was calculated over 300 s duration epochs in each recording. We then calculated the mean of these dFs: in surface fish the mean dF was 44.89 Hz (sd = 26.64 over 172 epochs) while in cavefish the mean dF was 78.39 Hz (sd = 47.92 over 938 epochs) (Figure 4A). Mean dFs in cavefish are significantly greater than in surface fish (Wilcoxon rank sum, two-sided, *z* = 8.84, *p* ≪ 0.0001). These findings are consistent with a role of the JAR in maintaining larger dFs between most individuals, but may also simply reflect the greater density of fish that we observed at the surface site.

Interestingly, we routinely found nearby fish with sustained, lasting for hundreds of seconds, differences in electric field frequencies that were below 10 Hz (Figure 4B). In surface fish, there were 294 pairs with mean dFs of <10 Hz over their entire recording sessions, and of those 142 pairs had mean dFs of <5 Hz (out of 1,119 potentially interacting pairs across five recording sessions). In cavefish, there were 18 pairs of fish with mean dFs that were <10 Hz over entire recording sessions, of which 4 were <5 Hz (out of total 2,356 potentially interacting pairs across 14 recording sessions). Pairs of fish were often at distances <50 cm during these low dF encounters (Figures 4C–E).

### 3.6. Patterns of Movement in Surface and Cavefish

We estimated the position of each fish in and around the grid (Madhav et al., 2018) in our recordings. Cavefish appeared to swim within small regions or territories on the order of tens of centimeters in diameter (Figure 2B). In contrast, surface fish appeared to have widely overlapping swimming trajectories (Figure 2A). To examine the relative movements of fish, we divided the data into 300 s duration epochs and fitted a minimum convex polygon to each fish's positions.

Although there was no difference in the overall size of the convex polygons between surface and cavefish (Wilcoxon rank sum, two-sided, *z* = 0.28, *p* = 0.7822), we found significantly more overlap in the trajectories of surface fish. The overlap between convex polygons of pairs of surface fish (mean = 13.89% overlap, sd = 9.57, *n* = 1, 371 comparisons) was significantly greater (Wilcoxon rank sum, two-sided, *z* = 14.14, *p* ≪ 0.0001) than in cavefish (mean = 8.41%, sd = 9.08, *n* = 950 comparisons).

Our impression is that the cavefish are more territorial than surface fish, at least while the surface fish are feeding at night. The increased overlap in trajectories in surface fish may be a result of their lower amplitude electric fields, which could reduce the distance for detection of conspecifics, localized distribution of food resources in the substrate, or simply due to the larger numbers of surface fish at the study sites. We expect that the surface fish may defend territories during the day when hiding in refugia. The necessary grid recordings were not possible during the day—additional recordings are needed to explore the diurnal modulation of social behavior in the surface fish.

### 3.7. Correlations Between Movement and EOD Frequencies Suggest Envelope Responses in the Field

**Figure 5:**
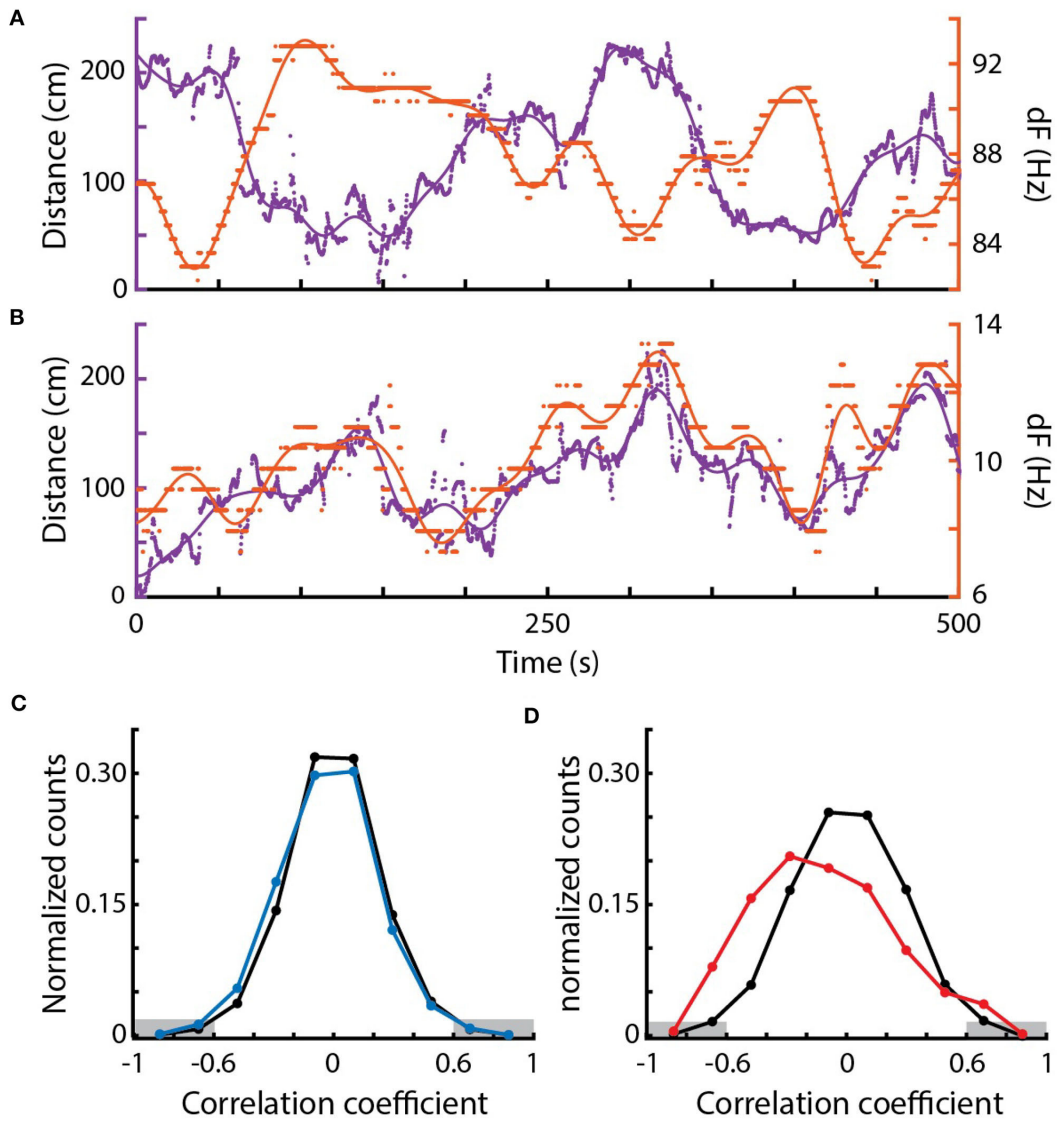
Correlations between EOD frequency and movement. **(A)** Example of a strong negative correlation between distance (purple, left) and dF (orange, right) of a pair of *Eigenmannia* over a period of 500 s. Dots are measurements, lines are low-pass fits of the data. **(B)** Example of a strong positive correlation. **(C)** Distribution of Pearson correlations between distance and dF for surface fish (blue) and shuffled data (black). **(D)** Distribution of Pearson correlations between distance and dF for cavefish (red) and shuffled data (black). There were significantly more strong Pearson correlations, below −0.6 and above 0.6 (marked in gray) in the original data when compared to the shuffled data in cavefish but not surface fish.

We examined the relations between relative movement and EOD frequency as fish interacted with conspecifics. We measured the dFs of all pairs of fish and their simultaneous pairwise distances over 300 s epochs (Figure 5). In many pairs of fish, dF and distance appeared to be strongly correlated—either changing in the same or opposite directions (Figures 5A,B). These correlations are likely “envelope” responses (Stamper et al., 2012, 2013; Metzen and Chacron, 2013; Huang and Chacron, 2016; Thomas et al., 2018). As *Eigenmannia* move closer together, the relative amplitudes of each fish's electric field increases (similar to moving closer to a sound source). Fish respond to these increases by shifting their electric field frequency either up or down (Metzen and Chacron, 2013; Stamper et al., 2013; Huang and Chacron, 2016; Thomas et al., 2018).

Correlations between two independently varying measurements may, however, occur spontaneously. To assess the rate of spontaneous correlations, we shuffled the distance and dF trajectories in time. We compared distributions of Pearson correlations between dF and distance in the shuffled and original data (Figures 5C,D).

In cavefish, we found both significantly more negative and positive correlations (Fisher's exact test, < −0.6: *p* = 0.00005, >0.6: *p* = 0.0107; 1,630 epochs). Interestingly, positive correlations were observed in fish with dFs of lower than 10 Hz (Figure 5B). Such positive correlations at low dFs are unexpected because of the impact of the JAR: the JAR is strongest at dFs of ~2–8 Hz (Heiligenberg, 1991) and should increase dFs with nearby fish, generally resulting in negative correlations between distance and dF. These unexpected positive correlations may be a result of the production of social signals that “override” the JAR, or may be driven by social interactions with other nearby fish that have higher dFs.

We also found examples of strong Pearson correlations between dF and distance at dFs over 50 Hz and distances of over 100 cm (Figure 5A) in cavefish. This is interesting because these distances were previously believed to be beyond the boundary of the animal's ability to detect such signals (Bastian, 1981; Nelson et al., 1997; Henninger et al., 2018). These “spooky” interactions at large distances have also been seen in other species of weakly electric fishes, which demonstrates that individuals can detect and respond to each other via weak modulations of their electric fields (Henninger et al., 2018; Raab et al., 2019).

In contrast, correlations between EOD frequencies of surface fish were not significantly different from shuffled EODs (Fisher's exact test, < −0.6: p=0.814, >0.6: *p* = 0.580; 577 epochs; Figure 5C). The lack of envelope responses in the surface fish may result from the high densities of fish: competing simultaneous interactions with multiple fish may have diluted the strengths of the pairwise measurements that we used. There may be context-dependent changes in envelope responses, such as an elimination of envelope responses during feeding, or these surface fish may simply not generate envelope responses.

## 4. Discussion

Weakly electric fishes rely on their electric fields for social interactions and localizing objects including prey, which reduce their reliance on visual cues for these functions. Electrogenic species, therefore, appear to be well-suited for life in caves in which there are no visual signals. However, electrogenesis is energetically costly; caves commonly have reduced food resources (Zepon and Bichuette, 2017; Pipan et al., 2018). As a first step in describing changes and adaptations for cave life in electrogenic fishes, we compared the electric behavior and locomotor movement of a population of troglobitic weakly electric fish *Eigenmannia vicentespelea* to a nearby population of epigean fish, *Eigenmannia trilineata*.

We found that the cavefish had stronger electric fields than the surface fish. The distributions of EOD frequencies was greater in the cavefish than in surface fish. These differences in EOD frequencies may result from differences in movement: the cavefish appeared to maintain territories whereas the surface fish did not at night. We also found a difference in the distribution of dFs between individuals. Cavefish had greater dFs than the surface fish. However, we found examples in both surface and cavefish in which pairs of nearby fish, within about 50 cm, maintained dFs of below 10 Hz for minutes. Finally, cavefish but not surface fish exhibited strong correlations between relative movement and dF—a behavior known as an envelope response.

### 4.1. Energetics

The EOD amplitudes of the troglobitic *Eigenmannia* were, on average, higher than the nearby surface fish. This may be in part due to body size: a previous report (Bichuette and Trajano, 2006) showed that the cavefish are generally larger than the surface fish. Further, our preliminary anatomical evidence from diceCT scans suggest that the electric organs of cavefish may also be relatively larger than in the surface fish. Irrespective of size, the energetic cost of generating electric fields is high, consuming up to one quarter of an individual's energy budget (Salazar et al., 2013; Markham et al., 2016). The fact that cave *Eigenmannia* produce such energetically costly electric fields suggests that sufficient food resources are available and accessible. The loss of eyes and pigment in these cavefish, therefore, is likely not under strong selection for energetic costs, but rather neutral selection (Jeffery, 2009).

Indeed, Gymnotiform fishes throughout the Amazon basin have relatively small eyes. Over the years, we have encountered many individual fish with missing or damaged eyes. Further, we routinely observe dense infestations of nematode parasites in the eyes of individuals from a related genus, *Apteronotus*. These anecdotal observations are consistent with the theory that Gymnotiform fishes rely more heavily on electric sensing than vision for survival and reproduction. Gymnotiform fishes, including the troglobitic weakly electric cavefish *Eigenmannia vicentespelea*, represent a unique opportunity to study evolutionary changes related to sensory perception and behavioral control.

### 4.2. EOD Frequencies

The distributions of EOD frequencies in both of these groups of *Eigenmannia* were not strongly bi-modal, which is similar to previous observations of *Eigenmannia* in Ecuador (Tan et al., 2005). Further, we were not able to identify any frequency-dependent signaling that might be correlated with sex. Sex differences in EOD frequencies are well-known in *Apteronotus* (Fugère et al., 2010; Raab et al., 2019) and *Sternopygus* (Hopkins, 1972), and there may be sex differences in EOD frequencies in *Eigenmannia* (Dunlap and Zakon, 1998).

There was a significant difference in the distributions of EOD frequencies between the cavefish and surface fish, but the meaning of these differences remains unclear. The larger range of frequencies seen in the cavefish may be due to sustained interactions related to territoriality—fish in adjacent territories may increase their dFs over time due to sustained stimulation of the neural circuitry that controls the JAR (Oestreich and Zakon, 2002, 2005).

Unexpectedly, we observed instances of sustained, low-frequency dFs in both surface and cavefish. In laboratory settings, in which artificial mimics of conspecific signals were presented to fish (Watanabe and Takeda, 1963; Heiligenberg, 1973), low frequency dFs activate the JAR, which results in higher dFs. For such experiments, fish are typically held in tubes or other restraints to reduce movement (Watanabe and Takeda, 1963; Hitschfeld et al., 2009). In the laboratory, low-frequency dFs have been shown to impair electrolocation (Heiligenberg, 1973; Ramcharitar et al., 2005). In the wild, fish could rely on JARs to avoid jamming, or could move further apart to lower the effects of jamming signals (Tan et al., 2005). There is some evidence, however, that fish actively match EOD frequencies or jam each other with similar EOD frequencies (Tallarovic and Zakon, 2002).

It seems unlikely that the surface fish were experiencing significant impairment of sensory function despite the ongoing low-frequency jamming: these fish were engaged in feeding on prey under the substrate in complete darkness. If the active electrosensory system was indeed impaired by the low-frequency jamming, it is possible that the fish were instead relying on their ampullary electroreceptors. Studies in *Apteronotus albifrons* demonstrates that fish may use ampullary receptors to detect exogenously generated electric fields for prey capture (Nelson and MacIver, 1999). In addition, elasmobranchs have also been shown to detect and discriminate signals from substrate-bound prey using passive electroreception mediated by ampullary electroreceptors (Kalmijn, 1971, 1982). Clearly, the relations between the JAR and jamming differ between laboratory and field settings, reflecting the richer social and environmental milieu.

### 4.3. Territoriality

Territoriality is a form of space-related dominance (Kaufmann, 1983). The most prominent function of having a territory is to provide the holder with a secured supply of resources. In the epigean streams outside of the Terra Ronca cave, food resources for *Eigenmannia* appear to be widely distributed in sandy substrates. Our guess is that the size and distribution of prey items precludes territorial defense of food resources. On the other hand, we expect to find evidence of territoriality during the day, as refugia likely vary in quality and are relatively small.

Why cavefish exhibit evidence of territoriality is unclear. There are no known predators of *Eigenmannia* in the cave, eliminating the value of protective refugia. Territoriality may occur as a result of uneven distribution of food resources in the cave, due to other physical features that impact the fish, or a consequence of plesiomorphic social and/or reproductive behaviors. Territoriality has been described in other genera, such as *Gymnotus* (Zubizarreta et al., 2020).

### 4.4. Spooky Interactions at a Distance

The strength of electric fields in water decay at a rate of approximately distance cubed (Henninger et al., 2018). As a result, the distances between fish determine the strength of the interaction of their electric fields: nearby fish will experience higher EOD voltages than from those of distant fish. Because the EODs of *Eigenmannia* are nearly sinusoidal, distance will have an effect on the amplitude of modulations caused by the summation of EODs: nearby fish will have large amplitude modulations near 100%, whereas distant fish will have far lower depths of modulation, below 10%. The relative movement between fish will cause concomitant changes in the strengths of EODs and depths of modulation that are proportional to distance. The changes are known as “envelopes”—the modulation of amplitude modulations. Envelope stimuli can elicit changes in electric field frequencies in *Eigenmannia* and other Gymnotiform species (Stamper et al., 2013; Thomas et al., 2018). In other animals, sensory envelopes are used in a wide array of behavioral contexts including speech perception (Ríos-López et al., 2017) and stereopsis (Tanaka and Ohzawa, 2006).

We found strong correlations between distance and pairwise differences in EOD frequencies at large distances of over 1.5 m and dFs of over 50 Hz. These results are similar to reports from field studies that examined other Gymnotiform species (Henninger et al., 2018; Raab et al., 2019). These field studies suggest that electric fish are far more sensitive to electrosocial stimuli than previously appreciated, requiring a reexamination of the neural systems for their perception.

Negative correlations between distance and dF are likely driven by the amplitude envelope of electrical interference patterns, producing JAR-like behavioral responses (Stamper et al., 2012). These findings show that laboratory studies of envelope responses are ecologically relevant (Stamper et al., 2012, 2013; Metzen and Chacron, 2013; Huang and Chacron, 2016; Thomas et al., 2018). Cavefish also exhibited significantly more positive correlations between distance and dF. These positive correlations may be aggressive signals in which fish actively jam each other (Tallarovic and Zakon, 2002).

Changes is dF may also be mediated by the simultaneous interactions of EODs of more than two fish (Partridge and Heiligenberg, 1980; Stamper et al., 2012). Our analyses were limited to pairwise interactions. It is likely that the changes in EOD frequencies that we observed included responses to features of electrosensory signals, including “social envelopes” (Stamper et al., 2012), that emerged as a result of interactions between three or more fish. Weakly electric fish have been shown to discriminate between envelopes that are generated in different contexts (Thomas et al., 2018). Importantly, pairwise analyses like those used above do not capture higher order group dynamics (Miller et al., 2013). Such undiscovered emergent dynamics may have dramatic influences on both the movements and EOD frequencies of individuals within aggregations of freely moving *Eigenmannia*.

## Data Availability Statement

The raw data supporting the conclusions of this article will be made available by the authors, without undue reservation.

## Ethics Statement

The animal study was reviewed and approved by Rutgers Newark Institutional Animal Care and Use Committee.

## Author Contributions

MB, DS, and EF conceived and executed the field research. NA, MM, RJ, and EF analyzed the electrical data. DS collected and analyzed the diceCT data. MM, RJ, and NC contributed to the design, use, and analysis of data from the grid system. All authors contributed to the preparation of the manuscript.

## Conflict of Interest

The authors declare that the research was conducted in the absence of any commercial or financial relationships that could be construed as a potential conflict of interest.

## Supplementary Material

Supplementary Video 1 There are two scenes in the video. Several *Eigenmannia trilineata* feed on prey below the substrate. We can see that the fish are separated by distances on the order of single body lengths, between 10 and 20 cm, while foraging. The fish are mostly oriented head-first into the flow of the water. Visible light is provided by a handheld flashlight shone from above. As we have seen at other study sites in the Amazon basin, weakly electric fish often show little or no reaction to flashlights during dark nights. In the second scene, a solitary fish swims by the camera. The tail shows clear evidence of regrowth, possibly from a predation event or an injury caused by a conspecific.

Supplementary Video 2 Eyeless *Eigenmannia vicentespelea* are seen swimming in tight groups under rocks, and as individuals along sandy substrates. Unlike the surface fish, the cavefish were disturbed by filming and retreated from the open areas where they were swimming into adjacent rocky outcrops. Social interactions include coordinated face-to-face swimming, which is likely mediated by their electric sense. The social interactions and movements of these fish are, to our eyes, as complex as those seen in sighted species of *Eigenmannia*. No foraging behavior similar to that seen in the surface fish was filmed.
